# Comparison the treatment of anterior inferior tibiofibular ligament anatomical repair and syndesmosis screw fixation for syndesmotic injuries in ankle fracture

**DOI:** 10.1186/s12893-023-01982-z

**Published:** 2023-04-10

**Authors:** Xuping Lin, Chengquan Tu, Weihuang Lin, Weina Xie, Xiaowei Guo, Qingjun Liu

**Affiliations:** 1 Department of Orthopedic Surgery, The Affiliated Dongnan Hospital of Xiamen University, Zhangzhou, 363000 Fujian Province China; 2 School of Medicine, Xiamen University, Xiamen, 361005 China; 3 Department of Orthopedic Surgery, Xiamen Haicang Hospital, Xiamen, 361100 China; 4 Department of Anesthesiology, The Affiliated Dongnan Hospital of Xiamen University, Zhangzhou, 363000 China

**Keywords:** Ankle fracture, Ligament anatomical repair, Syndesmosis screw, Syndesmotic injuries, Outcome studies

## Abstract

**Background:**

The fixation method of syndesmotic injuries in ankle fractures remains controversial. The goal of the study was to compare radiographic and clinical outcomes between anterior inferior tibiofibular ligament (AITFL) anatomical repair with syndesmosis screw fixation in syndesmotic injuries.

**Methods:**

We analyzed 62 patients who were treated with AITFL anatomical repair or syndesmosis screw fixation for syndesmotic injuries in an advanced teaching hospital between March 2016 and March 2019. Fixation was performed with AITFL anatomical repair in 30 patients (AAR group) and syndesmosis screw in 32 patients (SS group). Radiographic evaluations were the differences in mean anterior and posterior (A difference and P difference) tibiofibular distance between injured and uninjured ankle computed tomography (CT) scan at 6 months postoperatively. Clinical evaluation of patients was done using the American Orthopaedic Foot & Ankle Society (AOFAS) Ankle Hindfoot Score, the Olerud-Molander Ankle (OMA) score and visual analogue scale (VAS) score at 1, 3, 6 months and 1, 2 years postoperatively.

**Results:**

The A difference and P difference on CT was no differences (1.6 ± 0.8 mm, 1.3 ± 0.7 mm vs. 1.5 ± 0.7 mm, 1.2 ± 0.7 mm) between the two groups (All of *P* > 0.05). The AAR group had higher mean AOFAS score (65.6 ± 5.9, 82.3 ± 4.2, 87.6 ± 5.6 vs. 61.8 ± 5.2, 79.1 ± 4.0, 83.8 ± 4.9; *P* = 0.008, 0.003, 0.007) and higher mean OMA score (45.7 ± 8.7, 79.2 ± 6.5, 84.1 ± 5.3 vs. 40.4 ± 7.3, 74.8 ± 6.3, 80.3 ± 5.8; *P* = 0.012, 0.009, 0.010)) at 1, 3 and 6 months postoperatively. The AAR group had lower mean VAS scores (2.6 ± 1.2, 1.7 ± 0.7 vs. 3.4 ± 1.2, 2.2 ± 1.1; *P* = 0.018, 0.038) at 1 and 3 months postoperatively.

**Conclusions:**

The results of this study suggest that the AITFL anatomical repair technique could effectively improve ankle function during daily activity. Therefore, AITFL anatomical repair technique is expected to become a better fixation method for syndesmotic injuries.

## Introduction

The ankle syndesmotic is a micro-movement joint with three-dimensional motion formed by the ligament complex between the distal tibia and fibula. Approximately 1% to 18% of all ankle sprains and 13% to 23% of ankle fractures involve syndesmotic injuries [1, 2]. Syndesmotic is crucial for integrity of the ankle joint and thus for weight bearing [3]. Therefore, ignoring syndesmotic injuries will cause a series of problems including posttraumatic arthritis, chronic ankle pain, disability and instability. It is important to obtain anatomical reduction and restore the biomechanical characteristics of three dimensional micromovement for syndesmotic injuries [4].

Syndesmosis screw fixation is the most popular treatment option for ankle fracture combined with syndesmotic injury [5]. However, this method is a static fixation and becomes controversial currently because it has a high complication concern [6]. In addition, this method could lead to biomechanics alteration and micro-motion restriction of syndesmosis [7, 8], which may increase posttraumatic arthritis rate [7]. Furthermore, several drawbacks of syndesmotic screw fixation have been reported, such as malreduction, screw breakage and the need for screw removal [9, 10]. Therefore, flexible fixation has been advocated in more recent literatures, such as anterior inferior tibiofibular ligament (AITFL) anatomical repair technique, Kirschner wire fixation, suture button fixation and bioabsorbable screw fixation [11–13]. Although a few studies have described good clinical results of AITFL anatomical repair for syndesmotic injuries, there is insufficient evidence in radiographic outcomes still controversy on the treatment of combined injury of syndesmotic injury [14–16].

Therefore, this study aims to compare the radiological and clinical outcomes between AITFL anatomical repair with syndesmotic screw fixation in syndesmotic injuries. We hypothesized that AITFL anatomical repair can be an alternative treatment option for syndesmotic injuries following anatomical reduction and fixation of ankle fracture, obviating the need for syndesmotic screw fixation and thereby preventing potential complications.

## Methods

We reviewed the medical charts and radiographic images in all patients of ankle fracture combined with syndesmotic injury who treated with AITFL anatomical repair technique and syndesmotic screw fixation in department of orthopedic surgery between April 2017 and April 2020. The inclusion criteria for this study were as follows: (1) The subject is skeletally mature patient with a type B or C ankle fracture according to the Danis-Weber classification. (2) The subject demonstrates AITFL rupture diagnosed either radiographically or intra-operatively (intra-operative diagnosis was based on stress testing the syndesmotic under direct or radiographic guidance). (3) The subject has no history of previous severe ankle injury and does not have an ipsilateral lower extremity injury that would impede results. (4) The subject had operative repair by either syndesmotic screw fixation (SS) or AITFL anatomical repair technique (AAR). The exclusion criteria for this study were as follows: (1) The subject has an open ankle fracture. (2) The subject has a pathologic fracture. (3) The subject has an AITFL avulsion fracture. (4) The subject has neuropathic arthropathy and chronic syndesmotic injuries.

The 2-year follow-up was conducted by orthopedic surgeons and physiotherapists involved in the study. During the follow-up, 7 patients failed in follow-up. A total of 62 patients (62 ankles) were ultimately included in this retrospective study, 30 patients underwent the AITFL anatomical repair technique (AAR group) and 32 patients underwent syndesmotic screw fixation (SS group). No statistically significant differences were found in age, gender, time to surgery, mechanism of injury, fracture type, the time of syndesmotic reduction, postoperative incision drainage and hospitalization time between the 2 groups (All of *P* > 0.05). The demographic characteristics and relevant surgical data of all participants are presented in Table 1. The indication for AITFL anatomical repair technique or syndesmotic screw fixation was dependent on the experience and judgment of the orthopedic surgeon without standardization. All patients who underwent fixation with either AITFL anatomical repair technique or syndesmotic screw fixation by the same experienced surgeon. All the operations were performed by the same surgeon throughout the study. The study protocol was approved by our Hospital Health Sciences Research Ethics Board and signed informed consent with all patients.

**Table 1:** Baseline patient characteristics

	**ALL (*n* = 62)**	**AAR (*n* = 30)**	**SS (*n* = 32)**	***P value***
Age, y	43.7 ± 13.0	44.3 ± 13.4	43.1 ± 12.8	0.725
Sex, n (%)				0.793
Male	32 (51.6)	16 (53.3)	16 (50.0)	
Female	30 (48.4)	14 (46.7)	16 (50.0)	
Time to treatment, d	4.6 ± 1.4	4.7 ± 1.4	4.5 ± 1.5	0.590
Mechanism of injury, n (%)				0.765
Low energy injury	26 (41.9)	12 (40.0)	14 (43.8)	
High energy injury	36 (58.1)	18 (60.0)	18 (56.2)	
Fracture classification, n (%)				0.818
Weber-Danis B	34 (54.8)	16 (53.3)	18 (56.3)	
Weber-Danis C	28 (45.2)	14 (46.7)	14 (43.7)	
Time of DTS fixation, min	15.6 ± 3.5	14.9 ± 3.1	16.2 ± 3.7	0.137
Postoperative incision drainage, ml	59.8 ± 13.3	57.1 ± 13.2	62.3 ± 13.1	0.125
Hospitalization time, d	9.4 ± 2.5	9.1 ± 2.4	9.8 ± 2.6	0.287

Values are, n (%) or mean ± SD

*Abbreviations*: *AAR* AITFL anatomical repair group, *SS* Syndesmotic screw group

### Operative technique

Surgery was performed in a standardized manner according to AO principles with open reduction and underwent standard plate and screw fixation. Hook test or external rotation stress examination verifying syndesmotic injuries was then performed with the ankle in maximal dorsiflexion. Widening of the tibiofibular clear space (TCS) and medial clear space (MCS) of ≥ 2 mm was confirmatory [17]. Only those fractures demonstrating increased TCS and MCS ≥ 2 mm underwent syndesmotic reduction with either AITFL anatomical repair or syndesmotic screw fixation.

In AAR group, standard plate and screw fixation for ankle fracture. After ankle fracture fixation, an absorbable anchor (LUPINE®, Depuy Mitek) with partially absorbable anchor rope (Orthocord®, Depuy Mitek) was inserted into anterolateral aspect of distal tibia at level of 1.5–2 cm above tibia plafond [16]. Then the syndesmosis was anatomically reduced under direct vision and maintained with a clamp. Intraoperative fluoroscopy was applied to check the reduction. Afterward, anchor ropes were tied to the fibular plate or distal fibula with proper tension. After the reduction and reliable fixation of syndesmosis, the ruptured AITFL was anatomically continuous sutured by 2–0 absorbable suture (MONOCRYL®, ETHICON) in a tension-free circumstance [16]. After removal of the large clamp, reduction was verified with fluoroscopy including the TCS, MCS. Hook test or external rotation stress examination was performed again to examine the stability of the syndesmotic. Once we achieved a satisfactory reduction, the irrigation and suction drainage were performed and then the incision was closed sequentially.

In SS group, standard plate and screw fixation for ankle fracture. After bony fixation, under fluoroscopic guidance and direct vision, syndesmosis was reduced and maintained with a clamp. One 2.5-mm drill holes were performed approximately 2 cm above and parallel to distal tibia joint line (through a plate hole if present) from posteriorlateral to anterior-medial direction. 3 cortices were drilled and then one 3.5-mm cortical screws were inserted [18]. Then the ruptured AITFL was only explored but not repaired. After removal of the large clamp, reduction was verified with fluoroscopy including the TCS, MCS. Hook test or external rotation stress examination was performed again to examine the stability of the syndesmotic. Once we achieved a satisfactory reduction, the irrigation and suction drainage were performed and then the incision was closed sequentially.

### Postoperative management

**Fig. 1:**
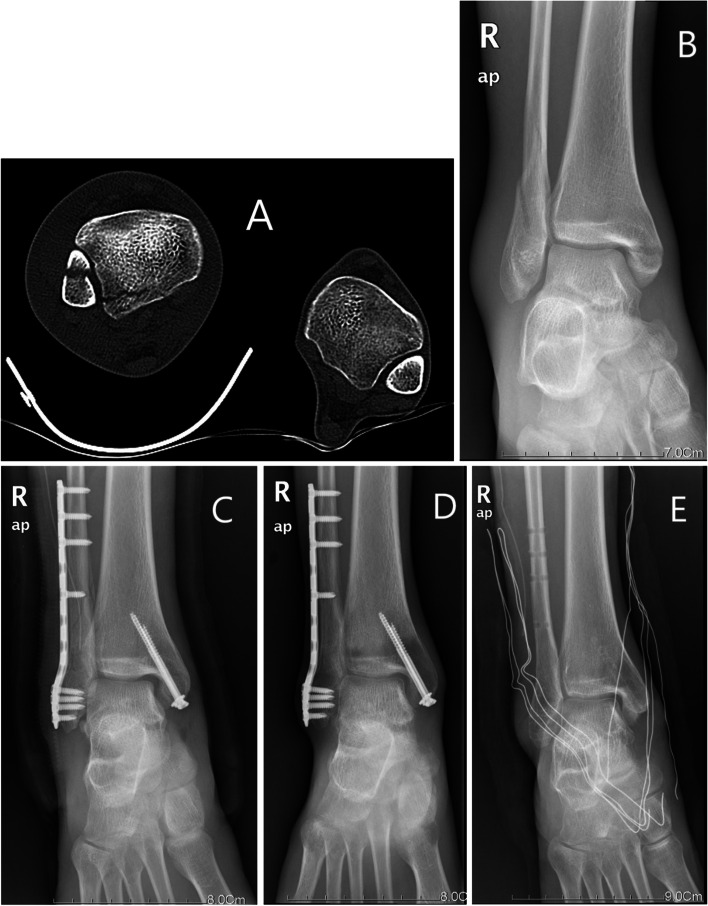
CT and X-ray images of AITFL anatomical repair. Notes: This patient underwent the AITFL anatomical repair technique. The radiolucent anchor and rope were not showed in the radiograph. **A** Preoperative CT image. **B** Preoperative radiographs image. **C** Radiograph at 1 days postoperatively. **D** Radiograph at 3 months postoperatively. Postoperative radiographs showed ankle fracture reduction maintained well. **E** Radiograph after removal of internal fixation

**Fig. 2:**
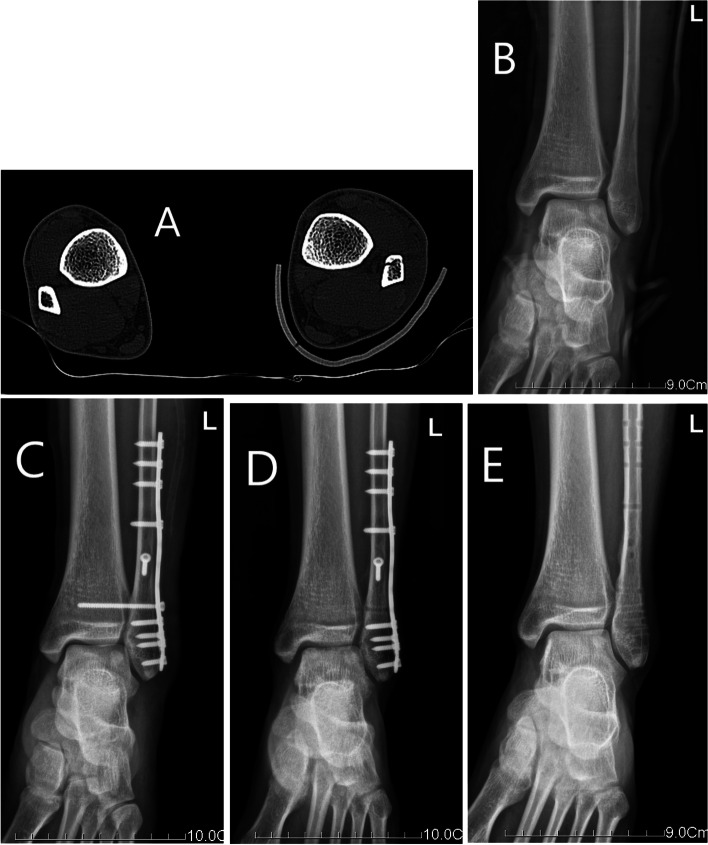
CT and X-ray images of syndesmotic screw fixation. Notes: This patient underwent the syndesmotic screw fixation. **A** Preoperative CT image. **B** Preoperative radiographs image. **C** Radiograph at 1 days postoperatively. **D** Radiograph at 3 months postoperatively. Postoperative radiographs showed ankle fracture reduction maintained well and the syndesmotic screw was removed. **E** Radiograph after removal of internal fixation

The ankle computed tomography (CT) scan, anteroposterior, lateral X-ray images were taken to investigate the reduction and implant location (Figs. 1 and 2). The wound sutures were removed after 2 weeks. The patients were advised to begin performing partial to full weightbearing rehabilitation after 6 weeks non-weightbearing postoperatively with active range of motion simultaneously [16]. The syndesmotic screw was removed in SS group at 3 months postoperatively, the plate and other screws were routinely removed in two groups at 1 year postoperatively. All patients were followed at 1, 3, 6 months, and 1, 2 years postoperatively.

### Radiographic evaluation

**Fig. 3:**
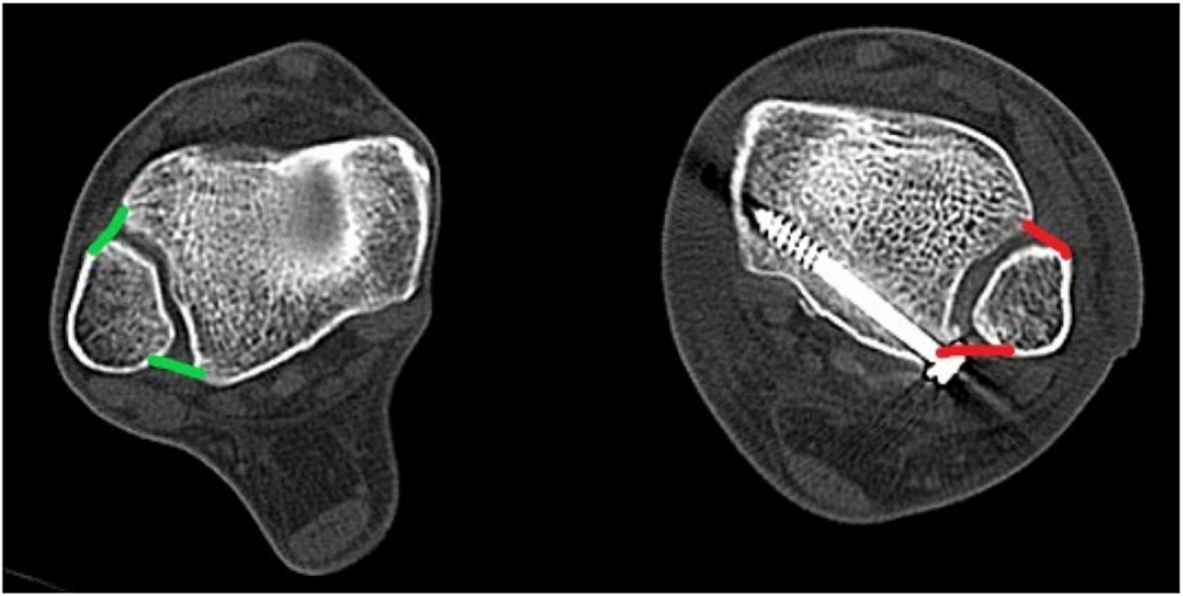
A difference and P difference were calculated as the distance between the anterior and posterior facets of the tibial incisura and the fibula. Notes: This patient underwent the AITFL anatomical repair technique. The operative side (red) is compared to the uninjured side (green) on computed tomography (CT) at 6 months postoperatively

There were 2 patients in the AAR group and 3 patients in the SS group failed to obtained ankle CT scan of health side. 57 patients underwent bilateral ankle CT scan, which used to assess the syndesmotic reduction at 6 months postoperatively [19]. Specifically, an axial cut 1 cm proximal to the ankle joint was used to determine the distance between the anterior and posterior facets of the tibial incisura and the fibula along a line perpendicular to the joint. The differences in mean anterior and posterior tibiofibular distance between injured and uninjured ankle on CT were primary radiographic evaluations (A difference and P difference) (Fig. 3). Malreduction was defined as a difference in syndesmotic width between normal and injured ankle of ≥ 2 mm [20]. Two investigators independently assessed anterior and posterior difference with an intraclass correlation coefficient (ICC) of 0.83 and 0.89.

### Clinical evaluation

Patients were evaluated at 1,3,6 months, and at 1, 2 years. The main outcome measure was the American Orthopaedic Foot and Ankle Society (AOFAS) Ankle Hindfoot Scale [21], ranging from 0 to 100 points, with 100 points being the best score. Secondary outcome measures included the Olerud-Molander Ankle (OMA) score, a self-administered patient questionnaire, ranging from 0 to 100, 100 being the best [22]. This score is evaluated against a linear analogue scale, the ability of ankle dorsiflexion while weight-bearing, OA, and ankle displacement on radiographs. Other secondary measure was visual analogue scale (VAS) a continuous scale for estimation of pain intensity, ranging from 0 to 10 where 10 is the limit for maximum pain [23]. VAS measures pain during rest, during, walking, at night, and during daily activities. In addition, complications were documented.

### Statistical analysis

SPSS statistical software package version 24.0 (IBM Corp., Armonk, NY) was used for statistical analysis. A Shapiro–Wilk test for normality was conducted for all continuous data, and the continuous data with the normal distribution was described in the form of mean ± standard deviation whereas the categorical data were described in number of cases (percentage). The age, time to surgery, time fixation of syndesmotic, postoperative incision drainage, hospitalization time, anterior and posterior difference, AOFAS score, OMA score and VAS score conformed to the normal distribution and the variance was homogeneous, expressed as x ± s. The continuous data with the normal distribution were analyzed by Student t test. As for the categorical variables, the chi-square test was performed. For all tests, the nominal type-1 error rate of 5% (*P* < 0.05) was considered the threshold for statistical significance.

## Results

A total of 62 patients were enrolled in the investigation. The mean age in all patients was 43.7 ± 13.0 years, in AAR group and SS group was 44.3 ± 13.4 years and 43.1 ± 12.8 years. The proportion of female patients was 48.4% in all patients, 46.7% in AAR group and 50.0% in SS group.

### Radiographic outcomes

**Fig. 4:**
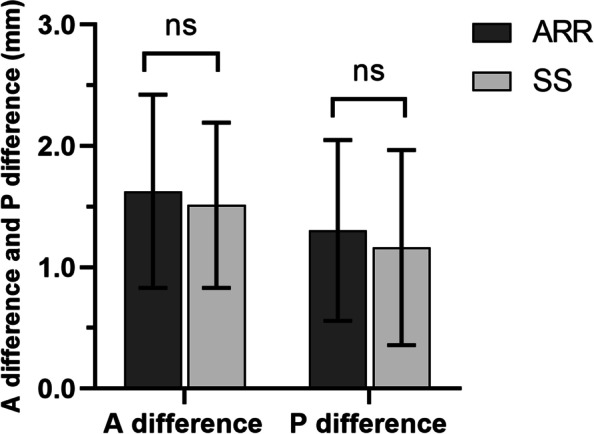
A difference and P difference comparison. Notes: The mean A difference and P difference were no significantly in the AAR group (1.6 ± 0.8 mm, 1.3 ± 0.7 mm) compared to the SS group at 1 year (1.5 ± 0.7 mm, 1.2 ± 0.7 mm) (All of *P* > 0.05)

On review of bilateral ankle CT scans at 6 months postoperatively, no differences in A and P difference (1.6 ± 0.8 mm, 1.3 ± 0.7 mm vs. 1.5 ± 0.7 mm, 1.2 ± 0.7 mm) on axial CT were noted between the two groups (All of *P* > 0.05) (Fig. 4). In addition, 6 patients in the AAR group (21.4%) and 8 patients in the SS group (27.6%) had a difference in syndesmotic width between normal and injured ankle of ≥ 2 mm.

### Clinical outcomes

All clinical assessments were administered at 1, 3, 6 months and 1, 2 years postoperatively.

#### AOFAS

The mean AOFAS score was significantly greater at 1, 3 and 6 months postoperatively in the AAR group (65.6 ± 5.9, 82.3 ± 4.2, 87.6 ± 5.6) compared with the SS group (61.8 ± 5.2, 79.1 ± 4.0, 83.8 ± 4.9) (*P* = 0.008, 0.003, 0.007). No differences in the mean AOFAS score were noted.between 2 groups at 1 and 2 years (All of *P* > 0.05) (Table 2).

**Table 2:** Clinical outcomes during follow-up

**Outcome Measure**	**AAR (*n *= 30)**	**SS (*n *= 32)**	***P *Value**
AOFAS score			
1 months	65.6 ± 5.9	61.8 ± 5.2	0.008
3 months	82.3 ± 4.2	79.1 ± 4.0	0.003
6 months	87.6 ± 5.6	83.8 ± 4.9	0.007
1 years	91.9 ± 3.1	90.1 ± 3.1	0.164
2 years	95.6 ± 3.0	94.2 ± 3.5	0.094
OMA score			
1 months	45.7 ± 8.7	40.4 ± 7.3	0.012
3 months	79.2 ± 6.5	74.8 ± 6.3	0.009
6 months	84.1 ± 5.3	80.3 ± 5.8	0.010
1 years	91.4 ± 4.0	90.5 ± 4.8	0.431
2 years	97.2 ± 3.1	95.7 ± 4.3	0.123
VAS for pain during daily activity			
1 months	2.6 ± 1.2	3.4 ± 1.2	0.018
3 months	1.7 ± 0.7	2.2 ± 1.1	0.038
6 months	1.1 ± 1.1	1.2 ± 1.0	0.850
1 years	0.6 ± 0.7	0.7 ± 0.8	0.456
2 years	0.4 ± 0.6	0.6 ± 0.7	0.173

Values are mean ± SD

*Abbreviations*: *AOFAS* American Orthopaedic Foot and Ankle Society, *OMA* Olerud-Molander Ankle score, VAS, Visual analogue score, *AAR* AITFL anatomical repair group, *SS* Syndesmotic screw group

#### OMA

The mean OMA score was significantly greater at 1, 3 and 6 months postoperatively in the AAR group (45.7 ± 8.7, 79.2 ± 6.5, 84.1 ± 5.3) compared with the SS group (40.4 ± 7.3, 74.8 ± 6.3, 80.3 ± 5.8) (*P* = 0.012, 0.009, 0.010). No differences in the mean OMA score were noted between 2 groups at 1 and 2 years (All of *P* > 0.05) (Table 2).

#### VAS

The mean VAS score was significantly lower at 1and 3 months postoperatively in the AAR group (2.6 ± 1.2, 1.7 ± 0.7) compared with the SS group (3.4 ± 1.2, 2.2 ± 1.1) (*P* = 0.018, 0.038). No differences in VAS score were noted between groups at 6 months and 1, 2 years. (All of *P* > 0.05) (Table 2).

There was 1 patient (3.1%) with a superficial infection and 1 patient (3.1%) with tissue irritation in the SS group, which resolved with antibiotic treatment. In addition, screws broke in 2 patients (6.3%), resulting in loss of reduction. In the AAR group, dehiscence was observed in 1 patient (3.3%) after suture removal, which healed after closing the skin again. 2 (6.7%) patient experienced loss of syndesmotic reduction in AAR group during the 2-year follow-up period.

## Discussion

This study compared the radiological and clinical outcomes between AITFL anatomical repair with syndesmotic screw fixation in syndesmotic injuries. Equivalent radiographic outcomes were observed between two groups, it suggests that AAR technique can maintain syndesmotic reduction as well as syndesmotic screw fixation. The clinical outcomes show improved ankle functional and lighter pain in AAR group at 1, 3 months, with higher AOFAS score, higher OMA scores and lower VAS score in AAR group.

We consider that AITFL repair provides syndesmosis stability at early stage, while the syndesmosis screw fixation is a rigid fixation which affects the mobility of the syndesmosis. Clanton et.al demonstrated the AITFL provides clincally significant stability to the syndesmosis, specifically providing resistance to posterior fibular translation and external rotation of the lateral malleolus when an external rotational force is applied [24]. However, long-term outcomes had no difference between the 2 groups. We attribute that the ruptured AITFL had recovered and the restoration of mobility of syndesmosis after removal of the screws. Due to AITFL anatomical repair can improved ankle functional in early, we believe the AITFL anatomical repair is prior to the syndesmotic screw fixation.

The ankle syndesmosis is mainly stable by syndesmotic ligament complex, in which AITFL and posterior-inferior tibiofibular ligament (PITFL) play the most important roles [25]. The AITFL provides the most stability against lateral displacement of the distal fibula and is the first ligament subjected to stress upon the application of external rotational force to the fibula [26, 27]. In most syndesmotic injuries, syndesmosis becomes unstable due to the ankle fracture and the rupture or dysfunction of AITFL [28, 29]. Once the ankle fracture is fixed well, the residual syndesmotic instability mainly results from the rupture of AITFL [28, 30]. In other words, AITFL anatomical repair can restore the stability of syndesmotic after fixation of ankle fracture. Recently, AITFL anatomical repair was increasingly being reported and has been shown in a biomechanical study to provide strength equal to or better than that of a normal ATFL [24, 31]. Therefore, we speculated that syndesmotic instability in ankle fracture with AITFL rupture could be treated by AITFL anatomical repair, while rigid fixation with regular syndesmotic screws would be unnecessary.

In this study, radiographic outcomes showed AAR technique can maintain syndesmotic reduction as well as syndesmotic screw fixation. Evaluation of a syndesmotic reduction has traditionally been performed using static and/or stress anteroposterior radiographs [32] and malreduction rates of 25%—52% have been reported in the literature with this technique [33]. However, these techniques are poorly defined and have been shown to be unreliable [32, 34, 35]. Currently, the most reliable radiologic method for establishing the true alignment of ankle mortise is postoperative CT [36, 37]. Bilateral CT investigations are suggested in the literature because of the possibility of individual or anatomic variations [38]. For this reason, bilateral CT scans at 6 months following operative intervention were used for assessment of reduction in the present study [39]. In addition, Andersen et al. [20] observed relationships between increased syndesmotic distance after surgery and poorer functional outcome, indicating that 2 mm difference can be used as a cut-off for revision surgery. We defined malreduction as a difference in syndesmotic width between normal and injured ankle of ≥ 2 mm [20, 40]. Our radiographic results agreed with two previous studies [30, 41] which showed that AITFL repair had an important part in maintenance of syndesmotic reduction and the repair of AITFL was a reliable fixation. Furthermore, Kee J et al. [5] shown that AITFL anatomical repair fixation has better radiographic outcomes and obviated the need for syndesmotic screw fixation in more than 80% of patients with syndesmotic instability.

AITFL anatomical repair technique is beneficial to the recovery of ankle function and relieve pain. AOFAS and VAS were chosen because of its widespread use. OMA has been validated against the Ankle Function Score and had a very high test–retest reliability [42, 43]. Yu Zhan et al. [16] shown that AITFL anatomical repair fixation has better ankle mobility with higher OMA scores and lower VAS scores for patients with syndesmotic injuries than syndesmosis screw fixation. Some studies [5, 14, 15, 39] reported that AITFL anatomical repair fixation had better clinical outcomes than syndesmosis screw fixation. This study also show that patients treated with AITFL anatomical repair had higher AOFAS, higher OMA scores, and lower VAS score than syndesmosis screw fixation. Beumer et al. [44] reported that the syndesmotic micro-motion of distal, anteroposterior movement and external rotation of the syndesmotic is essential for ankle function. Then we speculated that anatomical structure of AITFL was the key to achieve syndesmotic stability and AITFL repair can be used to restore the AITFL structure. This method could achieve dynamic syndesmotic stability immediately after operation with no restriction to syndesmosis micro-motion which could provide a dynamic support to have better recovery of ankle function and relieve pain [45].

There are several limitations to this retrospective study that are inherent in the study design, including the lack of randomization of treatment and the fact that the study was conducted in Chinese patients only, with a relatively small sample size. The inability to obtain weight-bearing CT imaging might be another limiting factor as syndesmotic may reduce under physiologic loads [39].

## Conclusions

In a word, AITFL anatomical repair technique can maintain syndesmotic reduction well and improve clinical outcomes in treatments of syndesmotic injuries. Thus, AITFL anatomical repair technique can make patients of syndesmotic injuries to restore their ankle functional quickly and better during daily activity. Therefore, AITFL anatomical repair technique is expected to become a better fixation method for syndesmotic injuries. Absolutely, there is no optimal treatment for syndesmotic injuries and further large-scale clinical studies may be required to explore better fixation methods.

## Data Availability

The datasets generated and analyzed during the current study are not publicly available due to the institution policy but are available from the corresponding author upon reasonable request.

## Abbreviations

AITFL: Anterior inferior tibiofibular ligament
A differences and P differences: The differences in mean anterior and posterior tibiofibular distance between injured and uninjured ankle computed tomography scan
CT: Computed tomography
AOFAS: American Orthopaedic Foot and Ankle Society Ankle Hindfoot Scale
OMA: Olerud-Molander Ankle
VAS: Visual analogue scale
TCS: Tibiofibular clear space
MCS: Medial clear space
ICC: Intraclass correlation coefficient
